# Molecular Assessment of Methylglyoxal-Induced Toxicity and Therapeutic Approaches in Various Diseases: Exploring the Interplay with the Glyoxalase System

**DOI:** 10.3390/life14020263

**Published:** 2024-02-17

**Authors:** Muhanad Alhujaily

**Affiliations:** Department of Clinical Laboratory Sciences, College of Applied Medical Sciences, University of Bisha, Bisha 61922, Saudi Arabia; malhujaily@ub.edu.sa; Tel.: +966-176238888

**Keywords:** methylglyoxal, glyoxalase 1, advanced glycation end products, diabetes, obesity, cardiovascular diseases, neurodegenerative disorders

## Abstract

This comprehensive exploration delves into the intricate interplay of methylglyoxal (MG) and glyoxalase 1 (GLO I) in various physiological and pathological contexts. The linchpin of the narrative revolves around the role of these small molecules in age-related issues, diabetes, obesity, cardiovascular diseases, and neurodegenerative disorders. Methylglyoxal, a reactive dicarbonyl metabolite, takes center stage, becoming a principal player in the development of AGEs and contributing to cell and tissue dysfunction. The dual facets of GLO I—activation and inhibition—unfold as potential therapeutic avenues. Activators, spanning synthetic drugs like candesartan to natural compounds like polyphenols and isothiocyanates, aim to restore GLO I function. These molecular enhancers showcase promising outcomes in conditions such as diabetic retinopathy, kidney disease, and beyond. On the contrary, GLO I inhibitors emerge as crucial players in cancer treatment, offering new possibilities in diseases associated with inflammation and multidrug resistance. The symphony of small molecules, from GLO I activators to inhibitors, presents a nuanced understanding of MG regulation. From natural compounds to synthetic drugs, each element contributes to a molecular orchestra, promising novel interventions and personalized approaches in the pursuit of health and wellbeing. The abstract concludes with an emphasis on the necessity of rigorous clinical trials to validate these findings and acknowledges the importance of individual variability in the complex landscape of health.

## 1. Introduction

### 1.1. The Glyoxalase System

**Figure 1:**
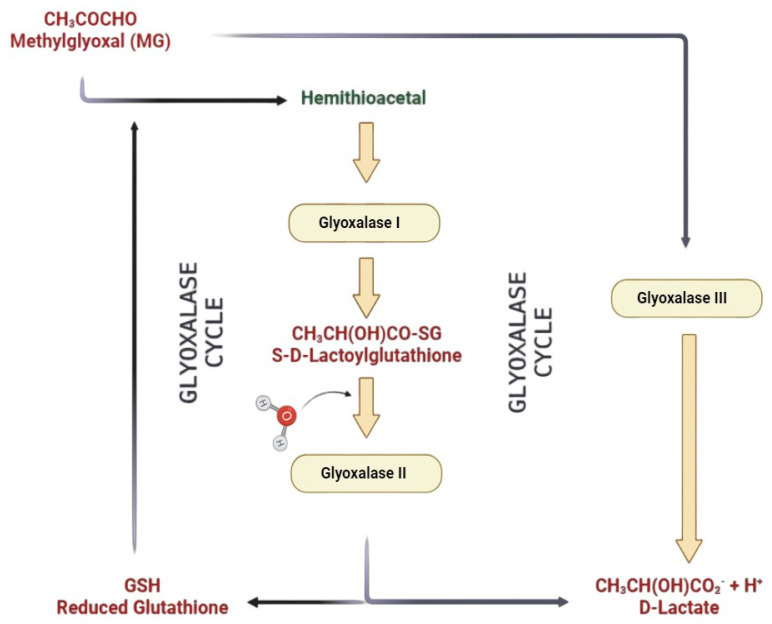
The glyoxalase system. The glyoxalase pathway consists of a two-step process involving GLO I and GLO II proteins. These enzymes facilitate the transformation of MG into D-lactate by utilizing GSH as a cofactor. In parallel, GLO III proteins perform a distinct one-step reaction, directly converting MG into D-lactate.

The glyoxalase system, a molecular maestro found in the cellular orchestra, orchestrates its performance in every mammalian cell’s cytoplasm and occasionally takes a bow in the nucleus of some cells [1]. This biochemical symphony stars two enzymes, GLO I and GLO II, accompanied by an enzymatic sidekick, reduced GSH. Together, they form a dynamic trio catalyzing the transformation of MG into D-lactate through the intermediate, S-D-lactoylglutathione, an essential act in the grand spectacle of cellular metabolism [2]. The overture begins with the spontaneous formation of hemithioacetal from MG in the presence of GSH. GLO I takes center stage, catalyzing the conversion of S-D-lactoylglutathione from hemithioacetal. Then, enter GLO II, the cleanup crew, S-D-lactoylglutathione hydrolyzing into D-lactate and reforming the indispensable GSH. This ballet of enzymatic precision ensures cellular harmony, with GLO I and GLO II performing in tandem as a system (Figure 1).

However, a twist in the plot emerges in prostate tumorigenesis. GLO I expression is seen in non-tumoral glands next to prostatic cancer, exclusively observed in luminal cells but shunning basal ones. On the malignant stage, 80% of prostate tissues exhibit modest GLO I expression. Surprisingly, manipulating GLO I—either knocking it down or amping up its expression—does not sway the expression of its partner, GLO II. This revelation hints at the separate functions of GLO I and GLO II (Figure 1) in the prostate tumorigenic drama [1]. The glyoxalase gene, a globetrotter in the biological realm, finds its home in a variety of biological systems, including protoctista, bacteria, fungi, yeasts, and humans [3]. Widespread distribution underscores its crucial role in biology. GLO I, a central enzyme, plays a pivotal role as a key rate-limiting factor. Dysregulation of GLO I emerges as a protagonist in serious human pathologies [4,5]. The script takes an intriguing turn as researchers delve into the regulatory nuances of GLO I. Gene copy number variation (CNV) and transcriptional control shape GLO I’s function. Small-molecule regulators of the glyoxalase system hold promise for uncovering biomolecular mysteries and developing pharmacological interventions in related diseases [6]. The glyoxalase system unveils a cellular narrative of balance and regulation, featuring an enzymatic duet and a catalytic companion, with potential therapeutic implications.

### 1.2. Glyoxalase I

The GLO I gene at 6p21.2 in humans exhibits diallelic diversity, encoding two akin subunits in heterozygotes. The resulting dimeric protein weighs 46 kDa (gel filtration) or 42 kDa (sequencing), with a pI dance between 4.8 and 5.1 [7,8,9]. This genetic saga reveals three human GLO I variants—GLO I-2, GLO I-3, and GLO II-2—representing the diallelic gene’s homozygous or heterozygous expression symphony on an autosomal locus [3,8].

GLO I, the metal-dependent maestro, carries the weight of a Zn^2+^ ion per subunit (in *E. coli*) or a Ni^2+^ ion in the human counterpart [10,11]. The human GLO I α AP-2 promoter region is a regulatory orchestration featuring players like AP-2α, E2F4, NF-κB, and AP-1. Amidst this genetic symphony, we find ARE, MRE, and IRE [5,9,12]. Notably, ARE also waltzes into GLO I exon 1, indicating a dance with Nrf2 [13,14]. Nrf2 activators, such as sulforaphane and resveratrol, take the role of GLO I conductors [15]. However, the exact details of this regulatory composition remain a melody to be fully composed. Current investigations explore the complex regulatory mechanisms of GLO1, involving gene expression and post-translational modifications. Acetylation and oxidation do not affect GLO1 activity, but glutathione acylation significantly impairs its functionality Currently, numerous investigations have delved into the intricate regulatory mechanisms governing GLO1. These mechanisms encompass both the regulation of gene expression and post-translational modifications of the enzyme. While acetylation and oxidation exhibit no impact on GLO1 activity, glutathione acylation significantly hinders its functionality [16]. Furthermore, GLO1 can undergo modifications such as phosphorylation at Thr107 and nitrosylation at Cys139 [17]. The regulation of GLO1 activity induced by TNF-α involves phosphorylation mediated by protein kinase A (PKA). This process leads to caspase-dependent cell death, accompanied by the generation of reactive oxygen species (ROS) [18]. ROS, in turn, dramatically inhibits GLO1 enzyme activity, resulting in the intracellular accumulation of the pro-apoptotic advanced glycation end product (AGE), argpyrimidine. This accumulation triggers a mitochondrial-dependent apoptotic pathway. Phosphorylated GLO1 plays a role in a pathway leading to the formation of specific methylglyoxal-derived AGEs. Additionally, the expression of GLO1 may be diminished by activating the receptor for advanced glycation end products (RAGEs), although the exact mechanism remains unclear. Moreover, the GLO1 gene serves as a hotspot for functional copy number variation (CNV), resulting in a 2–4-fold increase in GLO1 activity and expression.

The focus now turns to the complex regulatory mechanisms of GLO I, involving both modulation of gene expression and post-translational modifications. While acetylation and oxidation do not affect GLO I activity, glutathione acylation emerges as a potent inhibitor [16]. GLO I dons the attire of phosphorylation on Thr107, and TNFα orchestrates its modulation through PKA-induced phosphorylation, resulting in a dramatic ballet of caspase-dependent cell demise accompanied by a dazzling display of reactive oxygen species (ROS) [4,19,20]. These ROS spectacularly inhibit GLO I, ushering in the intracellular buildup of pro-apoptotic AGEs, such as argpyrimidine, and kickstarting a mitochondrial-dependent apoptotic pathway [21,22,23,24]. One important component of a process that produces particular MG-derived AGEs is phosphorylated GLO I [25]. Adding another layer to this genetic symphony, GLO I expression encounters modulation through the RAGE receptor being activated, though the specifics of this maneuver remain veiled [13,16]. Moreover, the GLO I gene plays host to a hot spot for functional CNV, causing a staggering 2–4 fold increase in both activity and expression [26,27,28]. And so, the genetic ballet of GLO I unfolds—a narrative of allelic diversity, metal-dependent nuances, and a regulatory symphony that orchestrates its crucial role in cellular harmony.

### 1.3. Glyoxalases II and III

GLO II, stationed at 16p13.3, emerges as additional key player in the glyoxalase narrative, encoded by the HAGH gene [10]. The human GLO II script unfolds across 10 exons, giving rise to two distinct mRNAs. One crafts the cytosolic GLO II (28.8 kDa), while the other weaves the mitochondrial GLO II (33.9 kDa), equally sharing an isoelectric point of 8.3 [8]. GLO II, a catalytic virtuoso, hydrolyzes S-D-lactoylglutathione, generating α-D-lactate and regenerating GSH with remarkable efficiency. This monomeric enzymatic maestro features a Fe(II)Zn(II) core and a metallo-β-lactamase-like α-helical domain. Intriguingly, Fe(II) has no influence on GLO II’s catalytic prowess, as observed in its graceful pirouette. The active site, staged in two domains, intricately interfaces the Fe(II)Zn(II) core and the substrate-binding location [12]. In the genetic script, intron 1 of human GLO II features a p53-responsive region activated by p63 and p73, leading to increased GLO II expression. This orchestrated symphony, directed by GLO II, plays a role in prostate tumorigenesis, influenced by the androgen receptor and the p53-p21 axis [1].

The plot thickens with PTEN loss, a herald of GLO I and GLO II upregulation. This rise to prominence is facilitated by the activation of the PI3K/AKT/mTOR pathway via the ERa axis and p-PKM2(Y105), ultimately propelling prostate tumor development [29]. Cells lacking GLO II find themselves at the mercy of MG or apoptosis triggered by DNA damage [8]. GLO II, a potent catalyst, warrants deeper exploration of its regulatory intricacies. GLO3, present in bacteria, suggests a human counterpart akin to a DJ-1 homolog, converting MG to α-D-lactate without GSH. The identity of human GLO3 remains elusive.

Shifting to pathology, dysregulated MG levels and a compromised glyoxalase system play a pivotal role in various diseases. Diminished system activity allows the accumulation of AGEs from MG, emerging as key players in age-related diseases. The interplay of MG and AGEs is crucial in diabetes, contributing to complications like nephropathy, neuropathy, and retinopathy. GLO I overexpression stands out as a guardian against diabetic nephropathy [30].

MG intertwines with atherosclerosis, attributed to low GLO I levels in plaques and AGEs like MG-H1 inducing LDL glycation and THP. Hypertension is linked to elevated MG levels, observed in rats with higher-than-normal MG amounts in kidney and aortic tissues [31,32]. In the realm of genetic maladies, KRIT1 (CCM1), associated with CCM, reveals a connection with GLO I, suggesting its involvement in CCM [5,33]. Neurodegenerative disorders become protagonists in the presence of AGE accumulation. GLO I finds itself entwined in the pathogenesis of these disorders.

On the contrary, anxiety disorders see an elevation in GLO I expression and a reduction in brain MG concentration, orchestrated by a decrease in the γ-aminobutyric acid pathway, a recognized anxiety mediator [34]. The tale extends to aging and epigenetics, fostering a tapestry of ailments [4]. GLO-I and GLO-II genes find expression in oocytes and cumulus cells in mice, with GLO-I playing a role in the viability of newborn Sertoli cells in pigs, casting them as pivotal actors in the intricate dance of infertility [35,36,37].

In cell death, MG emerges as a key player, while elevated GLO I expression and activity act as saviors for drug-resistant tumor cells with high glycolytic rates [27]. This positions GLO I as a potential target for antitumor drugs and a source of tumor diagnosis biomarkers, offering insights for chemotherapy strategies. The glyoxalase system, with its enzymatic ensemble, plays a central role in the drama of health and disease, woven with molecular intricacies and cellular symphonies.

Cells employ various detoxification mechanisms to prevent the harmful effects of MG. The glyoxalase system, a widespread enzymatic pathway in eukaryotic cells, serves as the primary defense against MG and other reactive dicarbonyl compounds [38]. This system, crucial for cellular protection against glycation and oxidative stress, involves two consecutive enzymatic reactions catalyzed by Glo-1 and Glo-2, with glutathione as a co-factor. Glo-1 converts the hemithioacetal produced by the non-enzymatic reaction of reduced glutathione (GSH) with MG into S-D-lactoylglutathione. Glo-2 then transforms S-D-lactoylglutathione into D-lactate, a less metabolizable enantiomer of L-lactate, recycling glutathione in the process [39]. As S-D-lactoylglutathione is non-toxic, the metabolism of MG by Glo-1 is a pivotal step in detoxification, indirectly influencing MG toxicity and advanced glycation end product (AGE) formation rates. Additionally, the recycling of glutathione during S-D-lactoylglutathione metabolism underscores the importance of maintaining balanced Glo-2 activity, as inadequate activity may lead to GSH trapping and reduced availability for other cellular processes, including defense against oxidative stress [40].

It is crucial to recognize that GLO1 activity is linked to GSH levels, which diminish under conditions such as oxidative stress. Unfortunately, GSH reduction also compromises the ubiquitin–proteasome system, reducing the cellular ability to degrade AGEs and handle glycation-derived damage [41]. This glyoxalase system is involved in metabolizing additional substrates like glyoxal, phenylglyoxal, and hydroxypyruvaldehyde. GLO1, being the rate-limiting enzyme, plays a pivotal role in preventing the accumulation of reactive α-oxoaldehydes, making it a key protector against glycative stress-induced AGE formation [42].

In the absence of glyoxalase activity, various systems, though not extensively explored, contribute to dicarbonyl detoxification. Alternative routes include aldehyde dehydrogenases (ALDHs), aldo-keto reductases (AKRs), DJ-1, and acetoacetate scavenging to form 3-hydroxyhexane-2,5-dione (3-HHD) [43]. AKRs, a large protein superfamily, reduce MG to hydroxyacetone or lactaldehyde, protecting against MG-induced cytotoxicity [44]. AKR1B3, studied in diabetic mice, showed increased MG and AGE levels [45]. Human studies indicated AKR1B1’s role in MG detoxification when GSH levels were low [46]. Schwann cells lacking glyoxalase activity exhibited increased AKR1B3 activity [47]. ALDHs, oxidizing MG to pyruvate, showed induced activity in response to MG treatment and in glyoxalase-deficient models [48]. Levels of 3-deoxyglucosone (3-DG), formed during sugar metabolism, were elevated in diabetic patients, with ALDH1A1 metabolizing it to 2-keto-3-deoxygluconic acid [49]. DJ-1 exhibited glyoxalase activity, preventing MG-induced damage and repairing MG-glycated proteins [50]. However, DJ-1’s contribution to MG accumulation was not observed in DJ-1 knockdown models [51]. Acetoacetate, a ketone body, non-enzymatically decreased MG levels during diabetic and dietary ketosis, demonstrating potential in preventing diabetes progression [52].

## 2. MG and GLO in Metabolic Syndrome 

Elevated levels of MG and a malfunctioning glyoxalase system are implicated in various diseases, particularly those associated with metabolic syndrome [53]. A compromised glyoxalase system, marked by reduced activity and expression, leads to the accumulation of MG and its byproducts, recognized as AGEs. This scenario is notably connected to age-related illnesses such as diabetes, heart problems, and neurological conditions [54].

The synthesis of MG and MG-derived AGEs is essential in the setting of diabetes for the pathogenesis of problems such as neuropathy, retinopathy, and nephropathy [55]. Studies in diabetic nephropathy have shown that reducing MG modification through increased expression of GLO I can prevent oxidative stress and mitigate kidney-related complications [30,56].

**Figure 2:**
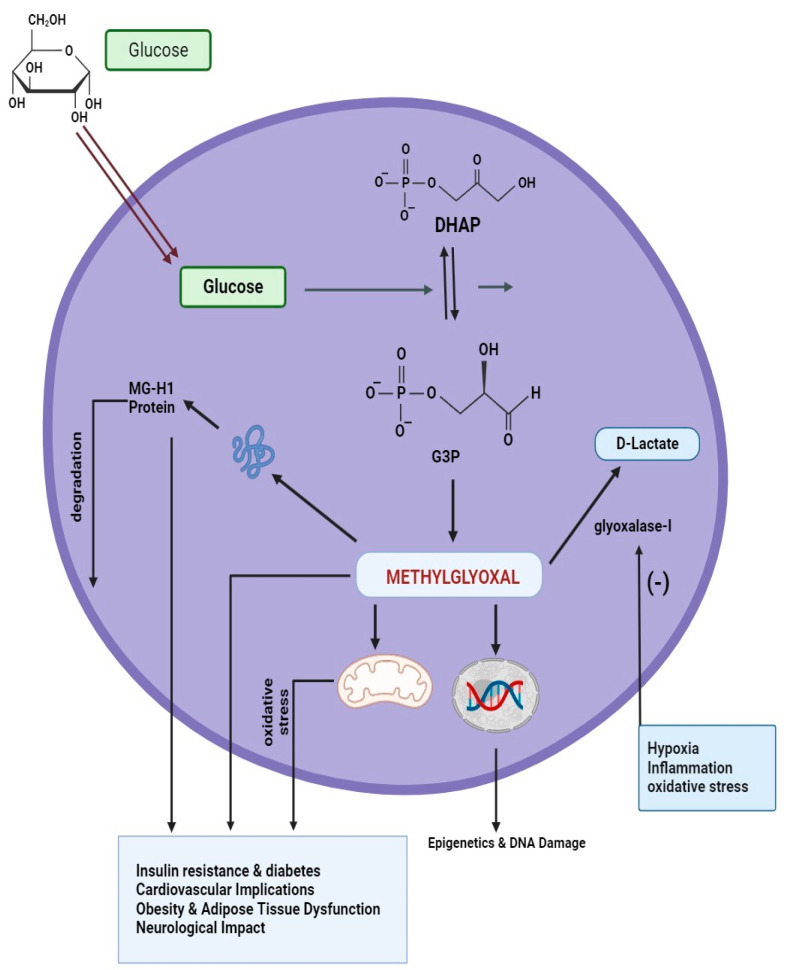
Comprehensive overview of MG and GLO system interaction in metabolic syndrome components. (G3P = glyceraldehyde 3-phosphate, and DHAP = dihydroxyacetone phosphate).

Cardiovascular diseases, specifically atherosclerosis and hypertension, are influenced by MG and GLO I (Figure 2) [57]. MG contributes to atherosclerosis via methods like low GLO I levels in ruptured plaques, the occurrence of MG-induced glycation, and AGEs produced from MG in atherosclerotic plaques of LDL [58]. The connection between MG and hypertension was evident in studies where elevated MG levels were observed in the kidney and aorta tissues of rats [59,60]. In the realm of neurodegenerative conditions, AGE buildup, driven by MG, affects brain function, contributing to conditions such as schizophrenia, Alzheimer’s, and Parkinson’s disease. GLO I’s involvement in these disorders underscores its role in maintaining cellular health [61].

Interestingly, in anxiety disorders, there is increased expression of GLO I, coupled with a reduction in MG concentration in the brain. This phenomenon is linked to decreased activation of the GABAA-receptor-GABA pathway, a known mediator of anxiety [34,62]. Aging and epigenetics further amplify the spectrum of diseases associated with MG and GLO I. The intricate roles of GLO I and GLO II genes in processes like infertility and cell death highlight their significance in maintaining overall health [5]. 

In the cancer context, the heightened activity of GLO I promotes the survival of cancer cells by decreasing MG levels. This underscores GLO I’s potential as a target for developing anticancer drugs and as a reservoir of biomarkers to diagnose tumors [63]. In essence, the interplay between MG and the glyoxalase system unfolds as a critical player in the complex landscape of metabolic syndrome-related diseases. MG and the glyoxalase system play intricate roles in the context of metabolic syndrome [64]. Here is a breakdown of their involvement:

### 2.1. Insulin Resistance (IR) and Diabetes

Elevated MG levels are associated with IR, a hallmark of metabolism-related syndrome and a precursor to type 2 diabetes. Dysregulation in the glyoxalase system, particularly reduced activity of GLO I, contributes to the buildup of MG. This, in turn, exacerbates IR and promotes the progression of diabetes, a key element of metabolic syndrome (Figure 2) [65].

### 2.2. Cardiovascular Implications

Metabolic disorder significantly surges the risk of cardiovascular diseases. MG, through its role in the development of AGEs, contributes to vascular damage. The glyoxalase system, particularly GLO I, has a vital part in mitigating this damage by detoxifying MG. Dysfunction in this system could exacerbate cardiovascular complications associated with metabolic syndrome [66] (Table 1).

### 2.3. Obesity and Adipose Tissue Dysfunction

Dysfunctional adipose tissue is a main characteristic of metabolic syndrome. MG has a part to play in the deterioration of adipocyte function and the development of obesity- associated complications. The GLO system, by regulating MG levels, may influence the health of adipose tissue and its contribution to metabolic syndrome (Figure 2) [67].

### 2.4. Oxidative Stress and Inflammation

Metabolic syndrome, marked by chronic inflammation and oxidative damage, may involve MG, a pro-oxidant molecule. The glyoxalase system serves as a defense, neutralizing MG to prevent its harmful effects. Impaired glyoxalase function could worsen inflammation and oxidative stress in metabolic syndrome [68]. 

Recent research has identified MG as a potent immunosuppressor. A study by Price et al. demonstrated that elevated MG levels inhibit T-cell proliferation, leading to reductions in both pro- and anti-inflammatory cytokines, including IFN-γ in myeloid cells and TNF-α and IL-10 in T cells [69] (Table 1). MG also diminishes the metabolic activity of myeloid-derived suppressor cells, a subset of regulatory immune cells with myeloid origin [70]. This immunosuppressive effect extends to CD8+ cytotoxic T cells, where MG transfer contributes to further immunosuppression [70]. Modification of histone H2A by MG increases immunogenicity, suggesting a potential role in autoimmune responses in cancer and the generation of autoantibodies [71].

In vitro studies on MG-AGE (methylglyoxal-derived advanced glycation end products) accumulation revealed that MG-AGEs impair inflammasome activation in macrophages, compromising innate immunosurveillance [72] (Table 1). This effect is independent of MG-AGEs binding to RAGE (receptor for advanced glycation end products) but results from the suppression of macrophage M1 polarization. M1 polarization, which induces a pro-inflammatory state conducive to phagocytosis, is hindered by MG-AGEs [72]. However, conflicting findings by Jin et al. suggested that MG-AGEs elevate RAGE expression in macrophages, subsequently triggering M1 polarization through NF-κB pathway activation [73].

### 2.5. Neurological Impact

Emerging research suggests that metabolic syndrome may have implications for neurological health. MG and AGEs, with their ability to induce oxidative stress, may contribute to neurodegenerative disorders often associated with metabolic syndrome. GLO I’s role in mitigating MG-related damage becomes crucial in maintaining neurological wellbeing(Table 1) [68].

**Table 1:** Interactions of MG and GLO I in metabolic syndrome-related diseases.

Disease Category	Associated Conditions	Role of MG	Role of GLO I	Implications and Findings	Reference
IR and Diabetes	Elevated MG levels, IR	Exacerbates IR, precursor to type 2 diabetes	Dysregulation leads to MG accumulation, worsening IR	Contributes to diabetes progression, central to metabolic syndrome	[65]
Cardiovascular Implications	Increased risk of cardiovascular diseases	Contributes to vascular damage through AGE formation	GLO I mitigates vascular damage by detoxifying MG	Dysfunction in glyoxalase system exacerbates cardiovascular complications associated with metabolic syndrome	[66]
Obesity and Adipose Dysfunction	Dysfunctional adipose tissue	Impairs adipocyte function, contributes to obesity-related complications	GLO I regulation of MG levels may influence adipose tissue health	MG and glyoxalase system play roles in adipose tissue dysfunction in metabolic syndrome	[67]
Oxidative Stress and Inflammation	Chronic low-grade inflammation, oxidative stress	Pro-oxidant role, contributes to oxidative stress and inflammation	GLO I acts as a defense mechanism, neutralizing MG to prevent damage	Impaired glyoxalase function could worsen oxidative stress and inflammation in metabolic syndrome	[68]
Neurological Impact	Implications for neurological health	MG and AGEs induce oxidative stress, contributing to neurodegenerative disorders	GLO I’s role is crucial in mitigating MG-related damage in the brain	Metabolic syndrome may have neurological implications, with MG and GLO I playing key roles in maintaining brain health	[68]

## 3. Roles of MG and Glyoxalase in T2D

When human RBCs were exposed to elevated glucose concentrations in short-term in vitro culture, the constant-state levels of S-D-lactoylglutathione and MG rose [7]. GLO I and GLO II activities, along with cellular GSH concentration, remained unchanged, while D-lactate concentration increased over incubation duration. Similar results were observed in human and cow aortic endothelial cells, as well as HMEC-1 cells in vitro. These findings, marked by elevated methylglyoxal (MG) production flux and steady-state concentrations, were linked to reduced GLO I activity, resembling conditions seen in hyperglycemia, a characteristic of diabetes [74]. Significantly, the excessive GLO I expression in endothelial cells grown with high glucose concentrations effectively barred the augmented AGE formation and reduced the development of tube-like structures. This reduction in tube-like structures represents impaired angiogenesis in the context of diabetes [75] (Table 2).

Cells with significant glucose uptake through the GLUT1 transporter, like mesangial cells, vascular endothelial cells, lens fiber cells, and pericytes, often exhibit elevated MG concentrations at steady state when incubated in high glucose concentrations [76]. This phenomenon arises from the heightened degradation of triosephosphates, DHAP, and GA3P, mainly through a step-in mammalian metabolism that is not enzymatic [77]. Increased cellular levels of GA3P and DHAP in hyperglycemia may result from reduced GA3PDH activity and heightened glucose consumption via anaerobic glycolysis. In this context, elevated MG concentrations may occur due to decreased in situ GLO I activity, influenced by reduced cellular GSH levels, potential glutathionylation, S-nitrosylation, and diminished GLO I expression [78].

Experimental diabetes research in STZ-induced diabetic mice revealed a surge in RBC GLO I activity and GLO II action, accompanied by a twofold increase in blood concentration of MG. Proteomic analysis further confirmed elevated GLO I protein levels in the glomeruli of diabetic rats compared to healthy normal controls [79]. In STZ-induced diabetic rats, a multifaceted response was observed in various tissues. This included reduced activities of both GLO I and GLO II in hepatic cells, while the activities of these enzymes were amplified in skeletal muscle [80]. The blood, lens, and renal medulla all had higher concentrations of MG. Blood levels of S-D-lactoylglutathione also rose. D-lactate expression levels displayed noticeable increases in the lens and blood [81]. These discoveries were consistent with observations in diabetic patients, where the levels of MG in plasma and red blood cells rose, and RBC GLO I activity was elevated. Notably, diabetic patients with microvascular complications exhibited elevated GLO I activity in RBCs compared to uncomplicated individuals, indicating a possible connection between increased GLO I activity and vascular complications in diabetes [82] (Table 2).

In diabetes, MG metabolism extends beyond the glyoxalase system, involving AKR, specifically aldose reductase. While aldose reductase contributes to MG metabolism, the glyoxalase system is expected to be the primary route in most human tissues under normal conditions, except for the renal medulla, which has unusually high levels of aldose reductase [83]. The function of the GLO system in MG metabolism is compromised under oxidative stress conditions, particularly when there is a decrease in GSH. In addition, GLO I may be inhibited through a process known as glutathionylation. Furthermore, GLO I expression has been downregulated in response to inflammatory signaling [82] (Table 2).

In pancreatic beta-cells, glyoxalase system-mediated MG metabolism influences insulin secretion. Elevated MG concentrations depolarized isolated rat pancreatic beta-cells, inducing increased cytosolic calcium concentration. While MG slightly and transiently stimulated insulin secretion, glucose-induced insulin secretion was suppressed [84]. The glyoxalase system’s presence in rat pancreatic islets was confirmed, with GLO I activity accounting for approximately 25% of that in the rat liver. However, the impact of GLO I inhibition on insulin secretion was weak, indicating a potential compensatory mechanism [85].

In protein modification, glyoxal and MG act as potent glycating agents, generating lysine-derived adducts (CML and CEL residues), bis(lysyl) crosslinks (GOLD and MOLD), and AGEs (G-H1 and MG-H1). These modifications can impact the degradation of AGE-modified proteins through cellular proteolysis and potentially contribute to vascular issues associated with diabetes [86,87]. 

**Table 2:** Overview of the key findings related to MG, the GLO system, and diabetes.

Aspect	Key Findings
In Vitro Exposure to Elevated Glucose	- RBCs exposed to high glucose show increased MG and S-D-lactoylglutathione levels.—GLO I and GLO II activities, GSH levels remain unchanged.—D-lactate levels increase over time [82].
Glucose Uptake via GLUT1	- Cells with predominant GLUT1 uptake exhibit increased MG levels in high glucose concentrations.—Heightened degradation of triosephosphates (GA3P, DHAP) through non-enzymatic processes [88].
Experimental Diabetes Studies (Animal Models)	- STZ-induced diabetic mice show increased RBC GLO I and GLO II activities, twofold increase in blood MG concentration.—Complex tissue-specific responses in STZ-induced diabetic rats [82].
GLO I Activity in Diabetic Patients	- Diabetic patients exhibit elevated MG levels in plasma and RBCs.—RBC GLO I activity is higher in patients with microvascular complications [89].
MG Metabolism Beyond Glyoxalase System	- AKR, particularly aldose reductase, plays a role in MG metabolism.—Glyoxalase system is the primary pathway in most human tissues, except in renal medulla [83].
Pancreatic Beta-Cell Studies	- MG impacts insulin secretion in pancreatic beta-cells.—MG induces depolarization, inhibits glucose-induced insulin secretion.—GLO I accounts for 25% of activity in rat liver [90].
Protein Modification and AGEs Formation	- Glyoxal and MG act as potent glycating agents, forming AGEs.—Impact on turnover of AGE-modified proteins, contributing to vascular complications in diabetes [55].

In summary, the intricate interplay of MG and the diabetes-related glyoxalase system involves alterations in MG concentrations, GLO I activity, and the formation of AGEs, impacting various tissues and contributing to the pathogenesis of vascular complications in diabetes. These findings underscore the complex nature of MG metabolism and its role in cellular dysfunction associated with diabetes.

## 4. Roles of MG and GLO in T2D Neuropathy

Diabetic neuropathy is a pervasive and challenging complication of diabetes, significantly impacting the quality of life of affected individuals [91]. Beyond the immediate discomfort and pain associated with neuropathy, it poses a considerable economic burden in diabetes care due to increased healthcare costs and potential disability. The syndrome encompasses both somatic and autonomic aspects of the peripheral nervous system, leading to a spectrum of symptoms that range from pain and tingling to numbness [91]. Alarmingly, approximately half of all individuals with diabetes experience some form of neuropathy, with a 15% risk of lower extremity amputation—an extreme consequence that underscores the severity of the condition [92].

Glycemic control, a cornerstone of diabetes management, is intricately linked to the progression of diabetic neuropathy [93]. Persistent hyperglycemia and oxidative stress play pivotal roles in initiating and perpetuating the damage observed in the nerves, Schwann cells, and microvasculature. The initial structural alterations in the microvasculature of peripheral nerves serve as the foundation for the sequence of events that ultimately result in neuropathy [94]. The role of glycation, specifically focusing on MG and glyoxalase, has emerged as a pivotal contributor in the development of diabetic neuropathy. Neuronal tissues, with their high energy demands and insulin-independent glucose absorption, are particularly susceptible to the deleterious effects of MG accumulation [66]. Studies have indicated elevated MG levels in sciatic nerves, especially in the context of STZ-induced diabetes [95,96]. Interestingly, higher MG levels have been identified as significant risk factors for the development of diabetic polyneuropathy, particularly in the early years following type 2 diabetes diagnosis. Preliminary investigations hint at a potential association between higher plasma MG levels and neurological pain in individuals with type 2 diabetes [97,98].

However, the complexity of these interactions is evident in conflicting results from larger studies, suggesting that serum MG levels might not be a precise reflection of MG concentrations in nervous tissue [99]. AGEs, such as MG-H1 derived from MG, have shown associations with the progression of neuropathy, particularly in type 1 diabetes. Insights from cell systems and animal models have further elucidated the importance of MG in diabetic neuropathy [100]. MG activation of signaling pathways in Schwann cells and its impact on neuronal cell viability underscore its significance. The glyoxalase system, particularly GLO I, is instrumental in detoxifying MG and plays a critical role in mitigating diabetic neuropathy. Diminished expression and activity of GLO I in diabetes contribute to increased MG concentrations and thermal hyperalgesia [79,101].

In diabetic neuropathy, unraveling the roles of MG and the glyoxalase system highlights targeting MG as a promising therapeutic approach. Strategies aimed at reducing MG levels, including novel peptide scavengers, hold potential for mitigating diabetes-induced hyperalgesia. Understanding these molecular mechanisms is crucial, offering avenues for therapeutic interventions to alleviate neuropathic symptoms and enhance overall diabetes management.

## 5. Roles of MG and GLO in Stroke

Stroke, a leading cause of morbidity and mortality globally, necessitates a comprehensive exploration of the intricate biochemical processes contributing to its pathogenesis [102]. MG, a metabolite derived from glucose metabolism, and the glyoxalase system, a critical defense mechanism against MG-induced damage, have recently emerged as key players in neurological health [103]. This section delves into the nuanced role of MG and glyoxalase in the context of stroke, shedding light on their potential implications for understanding and managing this complex cerebrovascular event.

Oxidative stress and neuroinflammation are hallmark features of stroke, significantly contributing to neuronal damage [104]. MG, known for its pro-oxidant properties, may exacerbate these conditions. Elevated MG levels have been observed in ischemic stroke, suggesting a potential role in amplifying oxidative stress and inflammatory responses [103]. Moreover, MG acts as a precursor in AGE formation, implicated in vascular complications. In the context of stroke, the accumulation of AGEs may contribute to vascular dysfunction and exacerbate the damage caused by the initial ischemic event [105]. 

The GLO system, composed of GLO I and GLO II enzymes, detoxifies MG. GLO I converts MG into S-D-lactoylglutathione, and GLO II further converts it to D-lactate. In heightened MG conditions, the glyoxalase system serves as a vital defense, preventing detrimental effects linked to MG accumulation. Upregulation of the GLO system is suggested to offer neuroprotection in stroke scenarios. By efficiently clearing MG, the glyoxalase system reduces AGE formation and lowers the risk of MG-induced neuronal damage.

In stroke management, the glyoxalase system emerges as a promising therapeutic target. Boosting glyoxalase activity through pharmacological interventions or lifestyle modifications holds potential to mitigate MG-induced damage and contribute to neuroprotection in stroke [106]. Further research is needed to assess the feasibility and efficacy of interventions targeting glyoxalase. Monitoring MG levels could provide insight into stroke diagnosis and prognosis, serving as biomarkers for oxidative stress and neuroinflammation. Integrating MG assessments into diagnostics may enhance our understanding of stroke subtypes and inform personalized treatment strategies.

Advancements in MG and glyoxalase system understanding pave the way for targeted therapies and precision medicine. Tailoring interventions to individual MG metabolism and glyoxalase activity profiles could revolutionize personalized stroke management. Combinatorial approaches addressing oxidative stress, neuroinflammation, and MG-induced damage may be promising. Integrating glyoxalase modulators with existing stroke therapies could enhance overall efficacy. The intricate interplay between MG and the GLO system unfolds a complex narrative in the context of stroke [107]. MG poses a dual threat, potentially intensifying oxidative stress and inflammation. The glyoxalase system acts as a guardian, shielding against MG-induced damage. Exploring the therapeutic potential of the glyoxalase system and understanding MG’s diagnostic significance opens new avenues in stroke research and management. Bridging scientific exploration with clinical application is crucial for advancing stroke care and enhancing outcomes for those affected by this formidable cerebrovascular event.

## 6. Roles of MG and GLO in CVD

**Figure 3:**
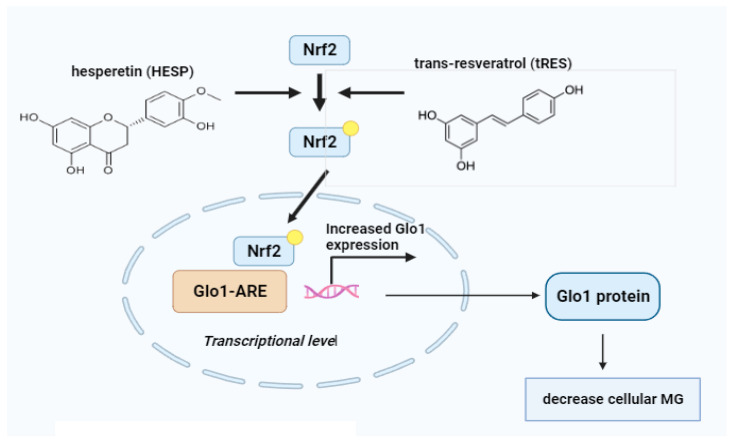
Schematic representation of the mechanisms of glyoxalase 1 (GLO1) regulation through the nuclear factor erythroid 2-related factor 2 (NRF2).

CVD stands as a formidable threat to individuals with diabetes, emerging as a principal cause of mortality. The key element underlying this risk is atherosclerosis, (atheromatous plaques formation within the walls of arteries). These plaques, consisting of a necrotic core and a fibrous cap, become particularly ominous when ruptured, leading to thrombus formation and heightened risk of critical CVD events such as myocardial infarction or stroke [108]. The intricate dance between diabetes and macrovascular complications unfolds through the formation of AGEs [109]. Within this context, the reactive metabolite MG takes center stage, earning attention for its potential in atherosclerosis onset. Studies directly administering MG have demonstrated a clear association with increased atherosclerosis, positioning MG as an active participant in the progression of this pathology [110]. Notably, MG has also been recognized as a predictor of intimal thickening in individuals with type 2 diabetes [111]. The modification of lipoproteins, specifically LDL and HDL, by MG further fuels the flames of atherosclerosis. MG’s modification of arginine residues in proteins, such as apolipoprotein B100, leads to the creation of MG-H1 and a more atherogenic LDL particle. Similarly, MG alters HDL, leading to decreased antioxidant and anti-inflammatory activities (Figure 3) [112].

Expanding the narrative, MG extends its influence to PDGFRβ, recognized as a target of MG modification. This modification may contribute to smooth muscle cell proliferation, potentially culminating in the development of rupture-vulnerable plaques [99]. Intriguingly, studies exploring GLO system function, specifically GLO I overexpression, yield conflicting results in mitigating atherosclerotic lesions in diabetic models (Figure 3) [82]. While GLO I overexpression does not consistently reduce lesion size, exposure to MG, either exogenously or through GLO I inhibition, has been demonstrated to augment atherogenesis. In addition to atherosclerosis, diabetes is intricately linked with impaired neovascularization, elevating the risk of mortality, post-ischemic events, lower limb amputations, and heart failure [79]. MG is an active player in this impairment, directly modifying HIF1α and reducing SERCA2a, resulting in defective new vessel formation and cardiac dysfunction, respectively (Figure 3) [99]. Beyond its implications in diabetes-related cardiovascular complications, MG significantly contributes to non-diabetes-related CVD. Two prominent cardiovascular complications associated with MG and its interplay with the glyoxalase system are atherosclerosis and hypertension [79] (Figure 3).

### 6.1. Atherosclerosis

**Figure 4:**
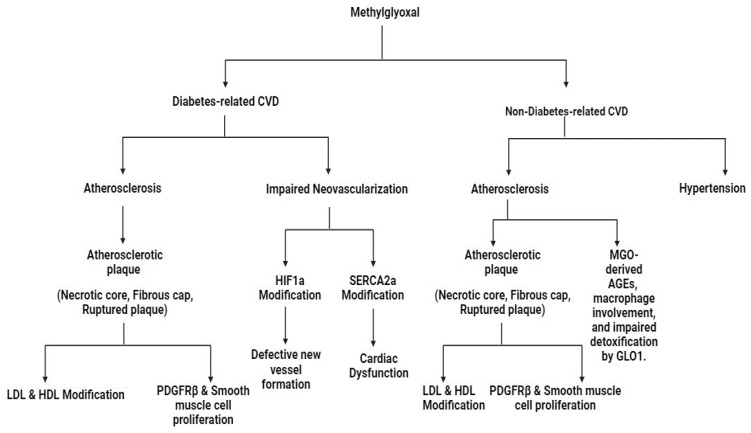
Schematic representation of the potential effects of Methylglyoxal in cardiovascular diseases.

MG’s influence on atherosclerosis extends to both diabetic and non-diabetic contexts. In atherosclerotic plaques, MG-derived AGEs, including MG-H1 and THP, are detected, with MG-H1 specifically linked to rupture-prone plaque phenotypes [113] (Figure 4). Notably, this association holds true regardless of diabetic status, highlighting the overriding impact of metabolic dysfunction within atherosclerotic plaques [113]. Macrophages in atherosclerotic plaques accumulate elevated MG levels due to heightened glycolytic activity and glucose uptake in the inflammatory and hypoxic environment. MG formation in macrophages is not solely from glycolysis but also results from lipid oxidation, connecting higher lipid content to increased MG-H1 levels [114]. MG modifies LDL, resulting in altered particle characteristics, heightened atherogenicity, and reduced clearance, thereby contributing to the progression of atherosclerosis (Figure 4). Within the complex milieu of atherosclerotic lesions, MG co-localizes with markers of apoptosis, hypoxia, oxidative stress, and inflammation, indicating its multifaceted role [115]. Additionally, impaired detoxification of MG by the GLO system is observed in atherosclerotic lesions, with reduced levels of GLO I observed in ruptured plaques as compared to stable plaques. In essence, MG significantly influences atherosclerosis onset through both enhanced levels and compromised GLO I detoxification, positioning it as a potential therapeutic target for comprehensive intervention [79].

### 6.2. Hypertension

MG’s influence extends to hypertension, a major global health threat. Studies in rats demonstrated enhanced MG levels in aortic and renal tissues, correlating with elevated systolic blood pressure [64]. Administration of MG to rats induced a significant rise in BP and increased plasma levels of catecholamines, angiotensin, aldosterone, and renin. Rat studies indicated that diets rich in fructose (MG precursor) led to the development of hypertension and renal injury [116]. Notably, the induction of hypertension by MG is observed in conditions of enhanced renal oxidative stress. Mechanistically, MG-induced hypertension involves the AT1-R-mediated pathway and enhanced cytosolic calcium levels, contributing to vascular retention [64]. Aminoguanidine, an MG scavenger, shows promise in mitigating MG-induced hypertension in rats. Clinically, multiple regression analysis identified MG as an independent risk factor for the elevation of systolic BP over a 5-year period in humans [117]. This underscores the potential utility of MG as a therapeutic target or biomarker for managing hypertension.

## 7. Roles of MG and GLO in Obesity

Obesity, now reaching epidemic proportions, plays a significant role in the rising prevalence of type 2 diabetes and CVD. Various factors associated with obesity contribute to the augmented formation of MG in obese individuals [118,119]. Studies in obese Zucker rats revealed the buildup of MG in adipose tissue, accompanied by increased serum MG levels [120]. Experiments on cell proliferation have demonstrated that straight incubation of adipocytes with MG leads to enhanced cell proliferation [120], suggesting the potential involvement of MG in the development of adipose tissue in obesity (Figure 4). In vivo studies involving long-term MG administration to normal rats (14 weeks) showed structural changes. Elevated tissue glycation and decreased apoptotic and angiogenic marker expression were linked to these effects, but not IR [121,122]. Conversely, short-term administration of MG for 8 weeks led to less severe effects, even though there was tissue accumulation of CEL [123].

In the context of obesity-related diseases, db/db mice (obesity model), exhibited lowered GLO I activity in the renal cortex [124]. Nonetheless, in RBCs of obese mice, there was a notable increase in GLO I activity by 50–60% when compared to their lean counterparts, and GLO II activity showed a corresponding rise of 20–30% [125]. Proteomic studies further revealed increased GLO I expression in rats (skeletal muscle) subjected to a high-fat diet [126]. Additionally, elevated transcription and GLO I enzyme activity were associated with mice preferring high-carbohydrate diets [127]. Conflicting findings highlight the need for further research to clarify GLO I’s precise role in obesity. Despite contradictions, these studies emphasize understanding MG and GLO I in obesity progression and adipose tissue dysfunction, suggesting potential implications for initiating type 2 diabetes (Figure 4). However, additional research is required to elucidate the underlying mechanisms and GLO I’s role in obesity.

## 8. Roles of MG and GLO in Cancer

Early research on MG revealed its antiproliferative impact, inhibiting DNA and protein synthesis, cellular respiration, and inducing DNA modifications associated with increased mutation frequency, strand breaks, and cytotoxicity [128]. MG’s toxicity selectively targeted proliferating and malignant cells, inhibiting cell growth in vitro, particularly in leukemia cells, while sparing mature peripheral leukocytes [129]. Additionally, MG treatment showed selectivity for mitochondrial respiration inhibition in malignant cells compared to normal cells [130].

MG demonstrated apoptotic induction in various cancer cell types, affecting cell cycle progression and glycolytic pathways [131]. It triggered the activation of MAPK family proteins and downregulation of Bcl-2 and MMP-9, impairing cell viability, proliferation, migration, invasion, and promoting apoptosis in cancer cells [132]. In liver cancer cells, lower MG concentrations reduced migration, invasion, and adhesion in a p53-dependent manner [133]. In vivo studies on rodents confirmed MG’s antitumor activity, inhibiting tumor growth, but limitations arose with tumor regrowth post-therapy cessation. The potential side effects of MG in chronic inflammatory conditions like obesity and diabetes remain unclear.

Cancer cells may exhibit survival mechanisms to counter high MG levels, such as increased Glo1 expression and activity. Glo1 overexpression has been associated with several cancers and multidrug resistance, presenting itself as a potential therapeutic target [134]. Amplification of the Glo1 gene is a common genetic event in various cancers, with higher expression observed in aggressive and invasive tumor cells. Silencing Glo1 inhibits tumor growth and induces apoptosis, suggesting its potential as a therapeutic target and prognostic indicator. In conclusion, MG and Glo1 play pivotal roles in cancer biology, influencing cell proliferation, survival, and apoptosis. Understanding these mechanisms may lead to targeted therapeutic strategies and improved prognostic assessments in cancer patients.

## 9. Roles of MG and GLO in Retinopathy

Limited information exists on the glyoxalase system’s role in ocular tissues, despite extensive research on non-ocular tissues. Emerging evidence suggests a connection between glyoxalase system alteration and diabetic retinopathy (DR). GLO1 and GLO2 expression is downregulated in DR patients, implicating a failure in the detoxification system [135]. In vitro studies indicate glyoxalase activity promotes pericyte survival under hyperglycemic conditions [136]. Transgenic rats overexpressing Glo1 showed inhibited retinal AGE formation and DR lesions were prevented, suggesting a potential therapeutic avenue [137]. Caution is warranted regarding expectations of GLO1 overexpression, especially in the lens. GLO1 is likely concentrated in the lens epithelium, where glucose is received from the aqueous or vitreous humor, potentially explaining age-related glycation-related browning, particularly in the lens core. As the efficacy of the glyoxalase system decreases with age, enhancing GLO1 activity could serve as a therapeutic strategy to counteract the accumulation of toxic compounds in the lens and retina.

However, caution is warranted in expecting GLO1 overexpression benefits, especially in the lens. GLO1 may primarily function in lens epithelial layers, potentially explaining glycation-related browning upon aging. Considering the decline in glyoxalase system efficacy with age, enhancing GLO1 activity might be a therapeutic strategy against toxic compound accumulation in the lens and retina.

Transcriptional modulators like metformin and candesartan, along with dietary compounds, exhibit GLO1-stimulating properties and show promise in mitigating glycation-related damage [138]. Further research is needed to explore nutritional and pharmacological approaches for DR associated with glycative stress.

## 10. Treatment Options with GLO I and MG; Therapeutic GLO 1 Inducers and GLO 1 Inhibitors

Dicarbonyl stress refers to the abnormal accumulation of dicarbonyl metabolites, causing heightened modifications of proteins and DNA. This phenomenon contributes to tissue and cell dysfunction in aging and various diseases. A crucial player in physiological systems is MG, a reactive dicarbonyl metabolite and major substrate for the GLO system [139]. In mammals, MG primarily forms through the spontaneous degradation of glycolytic intermediates [77]. The glyoxalase system, particularly GLO I, steps in to metabolize MG in the cytosol of all cells. GLO I facilitates the conversion of MG with reduced glutathione to S-D-lactoylglutathione, preventing the non-enzymatic reaction of MG with proteins and DNA [140]. However, when MG escapes metabolism, it can react with DNA and proteins, resulting in the formation of AGEs such as MG-H1 and MGdG. These AGEs are implicated in various metabolic and vascular diseases. MG-H1 formation in proteins leads to the substitution of charged hydrophilic arginine residue with hydrophobic uncharged MG-H1 residue. This process results in protein misfolding and triggers UPR [101].

Functional genomics studies on GLO I suggest that small-molecule GLO I inducers could have therapeutic potential. These benefits include preventing and reversing T2DM, treating microvascular complications in diabetes, and addressing conditions such as cardiovascular diseases, NAFLD, chronic kidney disease, age-related conditions, and respiratory issues [101].

Conversely, GLO I inhibitors are being explored for their potential in cancer chemotherapy and as adjunct therapies for malaria and MDR tumors. The reasoning behind this approach is to induce a substantial elevation in MG levels, selectively targeting rapidly proliferating malarial protozoa and tumors. This strategy exploits the observed selective toxicity of GLO I inhibitors toward tumors, as they tend to have higher glycolytic rates, increased MG formation, and often exhibit a hypoxic microenvironment [101].

The delicate balance of the glyoxalase system and its modulation using GLO I inducers and inhibitors present compelling avenues for therapeutic interventions in diseases linked to dicarbonyl stress. Exploring GLO I-inducing compounds, such as trans-resveratrol and hesperetin, along with GLO I inhibitors like BBGD, hints at the exciting potential of targeting this system for tailored treatment approaches [141].

### 10.1. Glyoxalase 1 Inducers

As the connection between increased MG formation and the development of dicarbonyl stress became understood as a critical factor in vascular complications of diabetes, efforts were made to decrease cellular MG concentrations through clinical strategies [65]. Early chemical scavenging agents, such as phenacylthiazolium bromide and aminoguanidine, demonstrated potency against MG but were discovered to be toxic or unstable [101]. A more promising approach involved boosting the expression and activity of GLO I, a catalyst in MG metabolism. GLO I induction was achieved through small-molecule activators of the transcription factor Nrf2, which binds to the GLO I genes ARE. The compounds tRES and HESP emerged as a potent combination, showing pharmacological synergism in activating GLO I-ARE transcriptional activity. 

This combination, referred to as tRES-HESP, exhibited significant effects on various cell types in vitro and demonstrated promising outcomes in pre-clinical and clinical studies [54]. In HAECs, tRES-HESP reduced the expression of receptors for RAGE and cell adhesion molecules, along with a decrease in inflammatory mediator secretion. In fibroblasts and a hepatoblastoma cell line (HepG2), tRES-HESP enhanced GSH cellular levels (Figure 3). The combination was also effective in experimental models, accelerating wound healing in diabetic mice and demonstrating anti-inflammatory effects [101].

The HATFF study with overweight and obese subjects receiving tRES-HESP orally for 8 weeks demonstrated high compliance and increased urinary tRES and HESP metabolite excretion. Notably, tRES-HESP raised GLO I activity in PBMCs by 22%, leading to a 37% decrease in MG plasma levels. This reduction was correlated with improved insulin resistance (IR) and reduced low-grade inflammation. Physiologically, tRES-HESP effectively corrected IR in overweight and obese individuals, restoring insulin sensitivity to levels observed in lean individuals. The combination also showed potential benefits in addressing dysglycemia, blood pressure, and dyslipidemia, surpassing the individual effects of tRES or HESP (Figure 3) [101]. The synergistic effects of tRES-HESP were attributed to improved bioavailability of tRES facilitated by HESP and pharmacological synergy in Nrf2 activation. In summary, tRES-HESP emerges as a promising dietary supplement warranting further clinical assessment, especially in the context of preventing and treating T2DM and the vascular complications linked to diabetes. Its well-tolerated nature and absence of reported adverse effects make it a viable candidate for chronic and prophylactic treatment applications, sparking ongoing evaluations [142]. 

### 10.2. Glyoxalase 1 Inhibitors

The development of GLO I inhibitors, particularly those that increase the concentration of MG, holds promise as a potential therapeutic avenue. The rationale for this approach stems from the understanding that GLO I inhibition leads to intracellular buildup of glyoxal and MG, which could be cytotoxic at elevated levels. While the initial exploration of GLO I inhibitors was rooted in anticancer drug development, there is emerging interest in their application in metabolic diseases [143]. Considering GLO I inhibitors as potential treatments for tumors, including TNBC, is relevant in the context of metabolic diseases. TNBC often exhibits elevated GLO I copy numbers and expression, with conventional chemotherapy showing limited effectiveness. Inhibitors targeting GLO I, which is associated with decreased patient survival, may provide a new therapeutic approach. Additionally, metabolic diseases like diabetes are linked with enhanced MG levels and dicarbonyl stress [144]. Strategies aimed at modulating GLO I activity could potentially alleviate dicarbonyl stress, which is implicated in the onset of vascular complications linked with atherosclerosis, CAD, and diabetes [145]. As research progresses, the development of GLO I inhibitors tailored to the unique metabolic challenges of diseases like diabetes could open new avenues for therapeutic interventions. By specifically targeting MG-associated pathways, these inhibitors may contribute to managing metabolic diseases and their associated complications.

Beyond its potential in diabetes and cancer therapy, cell-permeable GLO I inhibitors like BBGD have shown promise in combating pathogenic microbial infections [101]. In malaria caused by Plasmodium falciparum, BBGD demonstrated strong antimalarial activity in red blood cell cultures, inhibiting parasite nucleotide and protein synthesis. BBGD’s antiviral potential, especially against SARS-CoV-2, is also under exploration, motivated by historical reports of the antiviral activity of elevated MG concentrations, leading to an investigation into the SARS-CoV-2 proteome [146]. Analysis revealed significant enrichment of arginine residues, primary targets of MG modification, within functional domains of the SARS-CoV-2 proteome. This suggests vulnerability to functional impairment by MG, making GLO I inhibitors like BBGD potential antiviral agents. Ongoing research is assessing BBGD efficacy against SARS-CoV-2. BBGD serves as a valuable tool in studying pharmacologically-induced dicarbonyl stress, which is widely found in conditions such as atherosclerosis, diabetes, vascular complications, NAFLD, osteoporosis, age-related heart decline, and anxiety-linked behavior [101]. 

## 11. Small-Molecule Regulators of GLO 1

### 11.1. Small-Molecule Activators of GLO 1

When the GLO I enzyme is not as active as it should be, there is a buildup of MG, a key player in AGE formation. Mounting evidence indicates that increased levels of MG and the primary AGEs derived from it are associated with various age-linked issues like obesity, diabetes, cardiovascular diseases, and neurodegenerative conditions [4]. Thus, activating GLO I could be a promising approach to tackle these health problems related to aging. Previous research has identified different types of small molecules that can boost GLO I expression and activity, potentially preventing the buildup of MG and AGEs [147].

Candesartan, a synthetic drug, has the ability to stimulate GLO I. Research by Miller et al. in 2010 showed that candesartan restored the role of GLO I and release of nitric oxide in cells influenced by angiotensin II, which was related to retinal health in bovines. This is particularly significant in diabetic retinopathy, where candesartan’s GLO I-restoring function provides protective effects [148] (Table 3).

Moving on to natural compounds, polyphenols like resveratrol and fisetin enhance GLO I expression. Resveratrol, for example, enhanced the expression of HO-1 and GLO I, inhibiting oxidative stress and countering IR induced by MG [149]. The amalgamation of hesperetin and trans-resveratrol synergistically increased GLO I activity and reduced plasma MG levels, improving metabolic health in overweight and obese individuals [150].

Fisetin, another natural compound, boosted GLO I expression and activity while increasing the formation of its important co-factor, GSH, making it beneficial for treating diabetic patients [151] (Table 3). 

Mangiferin, a naturally occurring xanthone with C-glucoside, has been found to prevent diabetic nephropathy by enhancing GLO I function and inhibiting oxidative stress damage and the AGE/RAGE axis [152].

Isothiocyanates, present in cruciferous vegetables, act as activators of Nrf2, increasing GLO I activity and expression. Nrf2-ARE-GLO I pathway activation is crucial in defending cells against MG and AGEs derived from MG [153]. Bardoxolone methyl activated the Nrf2-Keap1-ARE pathway, potentially leading to an increase in GLO I expression and protecting kidney function in diabetic kidney disease [154]. Pyridoxamine, a natural vitamin B6 analog, induced GLO I expression and activity. It is considered a candidate for treating obesity and related inflammation and has shown promise in preventing retinopathy in diabetic rats. Notably, pyridoxamine not only slakes MG but also increases activity of GLO I [155] (Table 3).

**Table 3:** Overview of various small molecules that activate GLO I and their potential benefits in addressing age-related issues such as diabetes, obesity, cardiovascular diseases, and neurodegenerative disorders.

Small Molecule Activator	Type	Mechanism of Action	Associated Benefits
Candesartan	Synthetic Drug	Restores GLO I function and nitric oxide release affected by angiotensin II	Protective effects in diabetic retinopathy [148].
Resveratrol	Natural Polyphenol	Promotes HO-1 and GLO I expression, inhibits oxidative stress	Counters MG-induced IR, improves metabolic health [149].
Fisetin	Natural Polyphenol	Boosts GLO I activity and expression, increases GSH synthesis	Beneficial for treating diabetic patients, prevents diabetic nephropathy [151].
Mangiferin	Natural C-Glucoside	Enhances GLO I function, inhibits the AGE/RAGE axis and oxidative stress	Prevents diabetic nephropathy [152].
Isothiocyanates	Present in Cruciferous Vegetables	Activates Nrf2-ARE-GLO I pathway	Defends cells against MG and MG-derived AGEs [153]
Bardoxolone methyl	Synthetic Compound	Activates Nrf2-Keap1-ARE pathway	Protects function of kidneys in diabetic kidney disease [154].
Pyridoxamine	Vitamin B6 Analogue	Induces expression of GLO I and its activity	Candidate for treating obesity, prevents retinopathy in diabetic rats, quenches MG, increases GLO I activity [155]
Aminoguanidine, Alagebrium, Benfotiamine	MG Scavengers	Reduce MG levels, but mechanisms differ from those of GLO I activators	MG scavenging, prevention of MG-induced damage [4]

Lastly, MG scavengers reduce MG levels, but their mechanisms differ from those of GLO I activators. GLO I activators work by enhancing GLO I activity and expression in the GLO system to catalyze the change of MG to D-lactate. Pyridoxamine, as a unique case, not only reduces MG levels but also increases the activity of GLO I [4].

### 11.2. Small-Molecule Inhibitors of GLO

In tumors, GLO I overexpression supports cancer cell proliferation and survival by reducing intracellular MG levels and contributing to multidrug resistance. GLO I becomes a potential molecular target, providing an opportunity to improve the effectiveness of anticancer treatments. Recent evidence highlights the importance of finding GLO I inhibitors for treating diseases linked to inflammation, tumors, and multidrug resistance [63]. Various research groups are exploring different avenues, delving into drug research to pinpoint or craft powerful inhibitors for GLO I (Table 4).

Scientists are exploring different types of compounds to inhibit glyoxalase 1 (GLO I) as part of the quest for innovative antitumor drugs. One group of GSH-based compounds, which includes PBBG, showed potent inhibitory effects on GLO I [156]. However, the challenge was that such compounds could not easily penetrate cell membranes. To address this, derivatives like BBGC were developed. BBGC can efficiently cross cell membranes, inhibiting the growth of leukemia cells. Another set of inhibitors, S-(N/C-aryl/alkyl-N-hydroxycarbamoyl)glutathione derivatives, exhibited strong GLO I inhibitory effects. These inhibitors hold promise as targeted anticancer agents, showcasing potential selectivity as they serve as substrates for GLO II, which is more active in normal cells than in tumor cells. To enhance inhibition, researchers explored isosteric replacements and structural modifications. For instance, the compound CHG demonstrated competitive inhibition of GLO I, and its suberate diamide derivatives showed even stronger inhibitory effects [156].

Non-GSH inhibitors, including natural compounds like curcumin and flavonoids, have also been investigated. Curcumin, derived from turmeric, showed strong GLO I inhibitory effects [157] (Table 4). Flavonoids like myricetin and quercetin exhibited potent inhibition, with the OH group in the B ring contributing to effectiveness [158]. Moreover, flavonoid complexes found in nature with a ketone group at C-4 and OH group at C-5, such as delphinidin, demonstrated significant GLO I inhibitory activity [159]. Methyl-gerfelin, a derivative of gerfelin, also acted as a competitive GLO I inhibitor [160]. Among non-GSH inhibitors, NSAIDs, like acemetacin and indomethacin, and α-oxo-carbonic acids esters, such as ethyl pyruvate, showed inhibitory effects on GLO I [156]. N-hydroxypyridones and computer-aided drug design were also explored, identifying potent inhibitors like 4,6-diphenyl-N-hydroxypyridone and thiazolyl carboxylic acid derivatives [156]. The research encompasses a wide range of compounds, each contributing to the understanding and potential development of antitumor drugs targeting GLO I. 

**Table 4:** Overview of different small-molecule inhibitors targeting GLO I, showcasing their types, mechanisms of action, and potential benefits in the context of antitumor drug development.

Inhibitor	Type	Mechanism of Action	Benefits
PBBG	GSH-Based Compound	Potent inhibitory effects on GLO I	Effective GLO I inhibitor, but challenges with cell membrane penetration led to the development of derivatives like BBGC [156].
BBGC	GSH-Based Compound	Efficiently crosses cell membranes, inhibits leukemia cell growth	Improved membrane penetration compared to PBBG, potential for anticancer treatment [156].
S-(N/C-aryl/alkyl-N-hydroxycarbamoyl)glutathione derivatives	GSH-Based Compound	Strong GLO I inhibitory effects, potential selective anticancer agents	Substrates for GLO II, more active in normal cells, offering selectivity for tumor cells [156].
CHG	GSH-Based Compound	Demonstrates competitive inhibition of GLO I, suberate diamide derivatives show stronger effects	Effective competitive inhibitor of GLO I, with potential for enhanced inhibition [156].
Curcumin	Non-GSH Inhibitor	Strong GLO I inhibitory effects	Derived from turmeric, potential anti-tumor agent [157]
Flavonoids (myricetin, quercetin, delphinidin)	Non-GSH Inhibitors	Potent GLO I inhibition, hydroxyl group in B ring enhances effectiveness	Natural compounds with potential anticancer properties [158]
Methyl-gerfelin	Non-GSH Inhibitor	Competitive GLO I inhibitor	Acts as a competitive inhibitor of GLO I [160]
NSAIDs (acemetacin, indomethacin)	Non-GSH Inhibitors	Inhibitory effects on GLO I	Non-steroidal anti-inflammatory drugs show potential as GLO I inhibitors [156]
α-Oxo-carbonic acids esters (ethyl pyruvate)	Non-GSH Inhibitor	Exhibits inhibitory effects on GLO I	α-Oxo-carbonic acid esters as potential GLO I inhibitors [156]
N-Hydroxypyridones and thiazolyl carboxylic acid derivatives	Various Compounds	Identified potent inhibitors like 4,6-diphenyl-N-hydroxypyridone	Diverse range of compounds explored, contributing to the potential development of antitumor drugs targeting GLO I [156]

Metformin, a commonly used oral medication for type 2 diabetes, has diverse effects on GLO I according to various research reports. In diabetes treatment, metformin suppresses hepatic gluconeogenesis and enhances cellular glucose uptake. Its positive impact on heart-related issues in individuals with type 2 diabetes has been attributed to metformin potentially trapping MG [161,162]. Interestingly, metformin treatment not only reduces MG levels but is also correlated with a notable rise in the activity of GLO I within circulating cells. This dual action has led to the interpretation that metformin could function as a GLO I activator in treating type 2 diabetes (Table 4). Beyond diabetes, metformin demonstrates efficacy in other medical scenarios [161]. For instance, in terms of the correlation between diabetes and increased risks of endometrial cancer and hyperplasia, metformin has shown the ability to overcome progestin resistance. It achieves this by reducing the expression of GLO I and inhibiting activation of PI3K-mTOR, ultimately reversing progestin resistance. In the context of prostate cancer metastasis, metformin influences the suppression of the metastatic features of prostate cancer cells by inhibiting GLO I and promoting the expression of micro-RNA-101, a recognized cancer suppressor, which targets GLO I and decreases its expression [163]. Metformin-induced elevation of miR-101 and reduction of GLO I expression inhibited by GLO I-dependent control of EMT, a process linked to cancer progression. Furthermore, metformin enhanced the susceptibility of endometrial cancer cells to chemotherapeutic agents by suppressing the expression of GLO I [164]. This multifaceted impact positions metformin as a GLO I restorer, capable of reinstating normal GLO I activity and expression, thereby influencing various pathways in different disease contexts.

## 12. Conclusions

The quest for small-molecule regulators of GLO I and their impact on MG levels unfolds a compelling narrative in molecular regulation. Activating GLO I, with compounds like candesartan, polyphenols, isothiocyanates, and bardoxolone methyl, emerges as a promising strategy against MG havoc. On the flip side, GLO I inhibitors, such as PBBG derivatives, curcumin, flavonoids, NSAIDs, and metformin, are spotlighted in cancer treatment, offering potential against GLO I overexpression. This quest opens avenues in drug discovery, providing hope for inflammation and multidrug resistance-related diseases. The delicate balance between GLO I activation and inhibition holds the key to therapeutic innovation, promising interventions in age-related issues, diabetes, obesity, cardiovascular diseases, and neurodegenerative disorders. Substantiation through meticulous trials is crucial, acknowledging individual diversity and health contexts. The small compounds governing GLO I open possibilities for custom treatments and individualized strategies in the pursuit of optimal health and wellness.

## Abbreviations

Glyoxalase 1 (GLO1), Glyoxalase 2 (GLO2), Glutathione (GSH), Methylglyoxal (MG), Copy number variation (CNV), Isoelectric point (pI), Activator protein-2α (AP-2α), Early gene 2 factor isoform 4 (E2F4), Nuclear transcription factor-κB (NF-κB), Activator protein-1 (AP-1), Antioxidant-response element (ARE), Metal-response element (MRE), Insulin-response element (IRE), Insulin resistance (IR), Nuclear factor erythroid 2-related factor 2 (Nrf2), Advanced glycation end products (AGEs), Receptor for advanced glycation end products (RAGE), Hydroimidazolones N-(5-hydro-4-imidazolon-2-yl) ornithine (G-H1), N-(5-hydro-5-methyl-4-imidazolon-2-yl) ornithine (MG-H1), Hydroxyacylglutathione hydrolase gene (HAGH),, Red blood cells (RBCs), Glyceraldehyde 3-phosphate (GA3P), Dihydroxyacetone phosphate (DHAP), Glyceraldehyde-3-phosphate dehydrogenase (GA3PDH), Streptozotocin (STZ), Aldo-keto reductase (AKR), N-carboxymethyl-lysine (CML), N-carboxyethyl-lysine (CEL), Cardiovascular disease (CVD), Low-density lipoprotein (LDL), High-density lipoprotein (HDL), Human microvascular endothelial cell line (HMEC-1), Platelet-derived growth factor receptor β (PDGFRβ), Hypoxia-inducible factor 1α (HIF1α), Sarco(endo)plasmic reticulum Ca^2+^ ATPase (SERCA2a), Angiotensin II type 1 receptor (AT1-R), Unfolded protein response (UPR), Type 2 Diabetes Mellitus (T2DM), Trans-resveratrol (tRES), Hesperetin (HESP), Human aortal endothelial cells (HAECs), Health Aging Through Functional Food (HATFF), Peripheral blood mononuclear cells (PBMCs), Multidrug-resistant (MDR), Triple-negative breast cancer (TNBC), S-p-Bromobenzylglutathione diethyl ester (BBGD), Non-alcoholic fatty liver disease (NAFLD), S-p-bromobenzyl glutathione (PBBG), S-p-bromobenzyl glutathione cyclopentyl diester (BBGC), CHG (S-(N-p-chlorophenyl-N-hydroxycarbamoyl)glutathione), Epithelial-to-mesenchymal transition (EMT).
